# Making sense of the perceptual capacities in autistic and non-autistic adults

**DOI:** 10.1177/1362361320922640

**Published:** 2020-05-31

**Authors:** Jana Brinkert, Anna Remington

**Affiliations:** University of London, UK

**Keywords:** adults, attention, autism, perception, perceptual capacity, sensory processing

## Abstract

Recent studies highlighted that autistic individuals show increased perceptual capacity – the ability to process more information at any one time. This study examined whether there is a link between this increased perceptual capacity and the sensory hypersensitivity that many autistic people experience on a daily basis. In total, 38 autistic and 66 non-autistic adults filled in sensory questionnaires and performed an auditory load task, which assessed perceptual capacity. Results showed that higher levels of auditory perceptual capacity were correlated with higher levels of sensory sensitivities. We identified two clusters in the sample: one group of individuals with hyposensitivity and a decreased perceptual capacity (*n* = 42) and a cluster with an increased perceptual capacity and hypersensitivity (*n* = 47). Understanding this relationship may offer the opportunity to develop more effective techniques to ameliorate the often debilitating consequences of sensory hypersensitivity and over-arousal. Interestingly, this association between perceptual capacity and sensory sensitivities was seen for both groups; no significant association was found between perceptual capacity and level of autistic traits. As such, the findings may extend to other conditions with sensory atypicalities, such as attention-deficit hyperactivity disorder or Williams syndrome. The practical implications of the results for many aspects of daily life, education and employment are discussed.

**Lay abstract:**

Perceptual capacity refers to the amount of information that we can pay attention to at any one time. Research has shown that autistic people have a higher perceptual capacity, which means they can take in more information than non-autistic people can. This can be useful in certain situations, for instance, hearing approaching cars or noticing small details. However, in other situations, a higher perceptual capacity may result in more distraction. This study looked at whether having this increased perceptual capacity is linked to being very sensitive to sensory information (lights, sounds, touch, taste and smell) – something that many autistic people experience on a daily basis. Being very sensitive to these things can make it hard to interact with the world around us, so it is important to know more about what causes the sensitivity. To explore this, 38 autistic and 66 non-autistic adults completed a computer task that measured perceptual capacity and filled in a questionnaire about how sensitive they were to sensory information. We found that perceptual capacity was related to sensory symptoms for both autistic and non-autistic participants; people who had a larger perceptual capacity showed more sensitivity, while people who had a lower perceptual capacity showed reduced sensory sensitivity. This information can hopefully be used to improve the way in which we can support people who experience unpleasant sensory sensitivity.

A diagnosis of autism is based on social symptoms that not only affect communication and interaction with others but also non-social symptoms which include differences in behavioural patterns, interests and activities (American Psychiatric Association, 2013). The most recent publication of the *Diagnostic and Statistical Manual of Mental Disorders* (5th ed.; *DSM-5*) includes atypical sensory symptoms, such as hyperreactivity to sensory stimuli (the heightened acuity of sensory experiences) and hyporeactivity (an under-responsiveness to the sensory environment). Sensory processing is defined as the management of visual, auditory, tactile, proprioceptive and vestibular information that must be meaningfully interpreted to enable coherent daily life (Johnson-Ecker & Parham, 2000). Within this process, there is a distinction drawn between sensory sensitivity and reactivity. While many conceptual frameworks consider sensory perception to take a continuum from hypersensitivity to hyposensitivity (for a review see DuBois et al., 2017), other theories, such as the sensory integration theory (Dunn, 1997), include an additional behavioural dimension which distinguishes between reactive and passive responses to the sensory environment. In this study, we focus on hyper- and hyposensitivity (i.e. the threshold towards the sensory information) without implying a reactive behaviour response. In autism, sensory processing differences can affect all senses and can manifest themselves differently in every person (Tavassoli et al., 2014). Atypical sensory processing is not required for an autism diagnosis (*DSM*-5); however, this symptom is thought to be experienced by over 90% of autistic adults and children (Crane et al., 2009; Leekam et al., 2007) and persist throughout the lifespan (Kern et al., 2007).

These sensitivities can have a profound impact on daily life; sensory symptoms can be distressing, anxiety provoking and turbulent which can lead to withdrawal from sensory-rich environments as a coping mechanism (Jones et al., 2003). Indeed, sensory hypersensitivity has been described in autobiographical accounts as an overstimulation that is uncomfortable or painful similar to a ‘dentist’s drill hitting a nerve’ (Grandin, 1992, p. 1). It is therefore not surprising that sensory atypicalities are directly correlated with self-injurious behaviour (Soke et al., 2017). Interestingly, at the same time, sensory perception is reported by some autistic people as a source of pleasure and fascination (Jones et al., 2003), for example, the feeling of tin foil wrappers of chocolate, cold or metal surfaces or listening to music (Robertson & Simmons, 2015). More specifically, sensory processing has been reported as comfortable and pleasurable when control over the sensory source was possible. On the contrary, when participants were unable to control the sensory environment, sensory information was often reported as being uncomfortable (Robertson & Simmons, 2015).

Alongside the core symptoms discussed above, autistic individuals also often experience a different pattern of attention (e.g. Ames & Fletcher-Watson, 2010; Baron-Cohen et al., 2009). The reported atypicalities include both evidence for increased distractibility (Teder-Sälejärvi et al., 2005) and superior processing skills in fields, such as auditory perception (e.g. Järvinen-Pasley et al., 2008). This mixed set of observations may be explained by applying the load theory of attention and cognitive control (Lavie, 2005, 2010; Lavie et al., 2004). Load theory assumes that everyone has a certain ‘perceptual capacity’ (an amount of information that they can process at any given time) that must always be assigned in its entirety. If a task involves a great deal of information (high perceptual load), the full capacity may be allocated to task-relevant processing, leaving no surplus capacity to process additional task-irrelevant stimuli. When the perceptual load of a task is low (e.g. contains less information), one’s full perceptual capacity may not be required to complete the task. In this situation, any spare capacity will automatically process task-irrelevant information.

Our own, and others’, research has previously demonstrated that autistic people have increased perceptual capacity in the visual (Bayliss & Kritikos, 2011; Remington et al., 2009; Tillmann & Swettenham, 2017) and auditory domain (Remington & Fairnie, 2017). This higher capacity is beneficial when the task involves high levels of perceptual load, as it can be used to process task-relevant information and improve task performance. For example, on dual-task paradigms, autistic participants were better able to perform the central and secondary tasks simultaneously, even with high load on the central task, than their non-autistic peers (Remington et al., 2012; Remington & Fairnie, 2017). Conversely, having increased perceptual capacity may lead to higher susceptibility to distraction when performing a task with low perceptual load (as it will automatically result in task-irrelevant processing). For example, autistic participants showed an increased distractor interference effect under low levels of perceptual load compared to non-autistic participants when performing a letter-search task in the presence of distractors (Remington et al., 2009).

Increased capacity may, therefore, offer an explanation for a variety of phenomena experienced by autistic individuals, including – among others – pattern detection (Shah & Frith, 1993) and heightened pitch processing (e.g. Heaton et al., 2008; Mottron et al., 2006). The latter has been reported to be specifically evident in a subgroup of autistic people that is independent of intelligence or musical training (Heaton et al., 2008). In addition, other studies suggest that an increased distraction and a feeling of over-arousal due to processing multiple stimuli to fill the increased capacity (Remington et al., 2012). Indeed, autistic scholar Temple Grandin likened her auditory attention to a ‘. . . microphone that picks up all sounds with equal intensity’, but which also lead her to appear ‘deaf’ as she tries to shut out her sensory environment as a coping strategy in situations of auditory over-arousal (Grandin, 1995, pp. 67–68).

What is not yet known, however, is whether the extent to which someone has increased perceptual capacity is associated with their sensory sensitivities. This study, therefore, aimed to investigate whether increased perceptual capacity is linked to the everyday sensory experiences of autistic individuals. This is important given that daily sensory symptoms can be distressing for autistic people and have a profound impact on the ability to cope in social environments, school and the work place. It is therefore crucial to understand the mechanisms that may underlie this atypical sensory processing.

Previous theoretical accounts have alluded to a link between hypersensitivity and increased low-level information processing. For example, Baron-Cohen et al. (2009) suggested that sensory hypersensitivity is the basis for talent and attention to detail in autism. Similarly, the enhanced perceptual functioning theory suggests that atypical excitation and inhibition in primary sensory areas in the brain underpin observed increases in low-level perceptual function and superior processing abilities (Mottron et al., 2006). Dunn (1997) suggests that neurological thresholds of registration are directly related to sensory profiles.

To our knowledge, this study is the first to directly measure perceptual capacity and sensory sensitivity in a group of autistic and non-autistic individuals and investigate the relationship between the two variables. Furthermore, this research will elucidate whether any associations are unique to those on the autistic spectrum and are related to the level of autistic traits experienced. Understanding these relationships may help develop an approach to adapting the environment based on individual needs to minimise the negative impact of increased perceptual capacity while harnessing the benefits. This could have important and far-reaching consequences for everyday life, social interactions and learning.

## Method

### Participants

In total, 104 autistic and non-autistic adults, aged between 18 and 55 years, took part in the study. The participants were recruited through social media platforms, researchers’ own networks, advertisements around the University College London (UCL) campus and through the UCL research subject database. All autistic participants (*n* = 38) had previously received a clinical autism diagnosis from a trained and independent clinician (American Psychiatric Association, 2013). As the study involved auditory stimuli, participants’ audiometric air conduction thresholds were tested (in line with the British Society for Audiology, 2011) between the frequencies of 250 and 8000 Hz using a Kamplex Diagnostic Audiometer AD17 and Telephonics TDH-39-P headphones. All participants had normal hearing in both ears with a threshold better or equal to 15 dB HL.

Three participants in the autism group were excluded as their IQ levels were below 80 and one additional non-autistic participant due to an incomplete IQ test. Five further participants were excluded from each group due to low levels of accuracy (below 60%) on the auditory load task (see ‘Results’ section for more details).

### Ethics

The study was approved by the Department of Psychology and Human Development at the UCL Institute of Education, and all procedures were in accordance with the code of ethics of the British Psychological Society. Written informed consent was obtained from the participants prior to their participation.

### Measures

#### Social Responsiveness Scale

The adult self-report Social Responsiveness Scale, Second Edition (SRS-2; Constantino & Gruber, 2012) was completed by all participants. The SRS-2 is a 65-item questionnaire that assesses aspects of social behaviour. Scores are obtained for five subscales: Social Awareness, Social Cognition, Social Communication, Social Motivation, and Restricted Interests and Repetitive Behaviour, along with an overall score. Higher scores indicate greater social impairment, and the SRS-2 is widely used as a measure of autistic traits. A score over 60 indicates the clinical threshold for the autism diagnosis. The scale has excellent test–retest reliability (0.88–0.95), interrater reliability of 0.61–0.92 and good internal consistency (α = 0.95, Bruni, 2014).

#### Wechsler Abbreviated Scale of Intelligence

The Wechsler Abbreviated Scale of Intelligence, second edition (WASI-2, Wechsler, 2011), Full-Scale IQ-2 (FSIQ-2) was used to provide a measure IQ for each participant.

#### Sensory Perception Quotient

Participants completed the shortened Sensory Perception Quotient (SPQ; Tavassoli et al., 2014), a 35-item scale, that covers aspects of sensory processing that include touch, smell, vision, hearing and taste. The SPQ was developed to specifically assess fundamental sensory experiences in autistic individuals without assessing affective and behavioural sensations. Questions include, for example, *I would be the first to hear if there was a fly in the room*. Responses were given on a 4-point Likert-type scale (1 = strongly agree to 4 = strongly disagree). Four items on the questionnaire were reverse coded. Low scores on the SPQ indicate sensory hypersensitivity. The SPQ shows excellent reliability (α = 0.93) and moderate concurrent validity (*r* = −0.49, *p* = 0.007) with the Sensory Over-Responsivity Scales (Schoen et al., 2008).

#### Autism Diagnostic Observation Schedule

A subgroup of the autistic participants (*n* = 20) took part in an Autism Diagnostic Observation Schedule (ADOS-2, Lord et al., 2012) to confirm the level of autistic symptomatology. The ADOS-2 is a semi-structured, standardised assessment that rates the participant’s language and communication, reciprocal social interactions, imagination and stereotyped behaviours and restricted interests in line with the diagnostic criteria of the *DSM*-5 (Lord et al., 2012). The ADOS-2 Module 4 has good sensitivity (estimates ranging from 80.3% to 89.1%) and specificity (estimates of 62.1%–90.9%; Hus et al., 2014). All autistic participants who took part in the ADOS met the clinical diagnosis for autism using the ADOS Module 4. For some participants, we were unable to perform the ADOS due to time constraints. Instead, the SRS-2 was used to verify the level of autistic traits. We recognise that this is a limitation of our approach; however, our aim was to examine autistic traits (through SRS-2 scores) as a continuous variable rather than perform group comparisons based on diagnostic classification.

#### Auditory load task

The auditory load task from Fairnie et al. (2016) was presented on a Toshiba Portégé R930-1D7 laptop in OpenSesame version 3.1.7 experimental software (Mathôt et al., 2012) through Audio-Technica ATH-M30X Professional Monitor Headphones.

**Figure 1.**
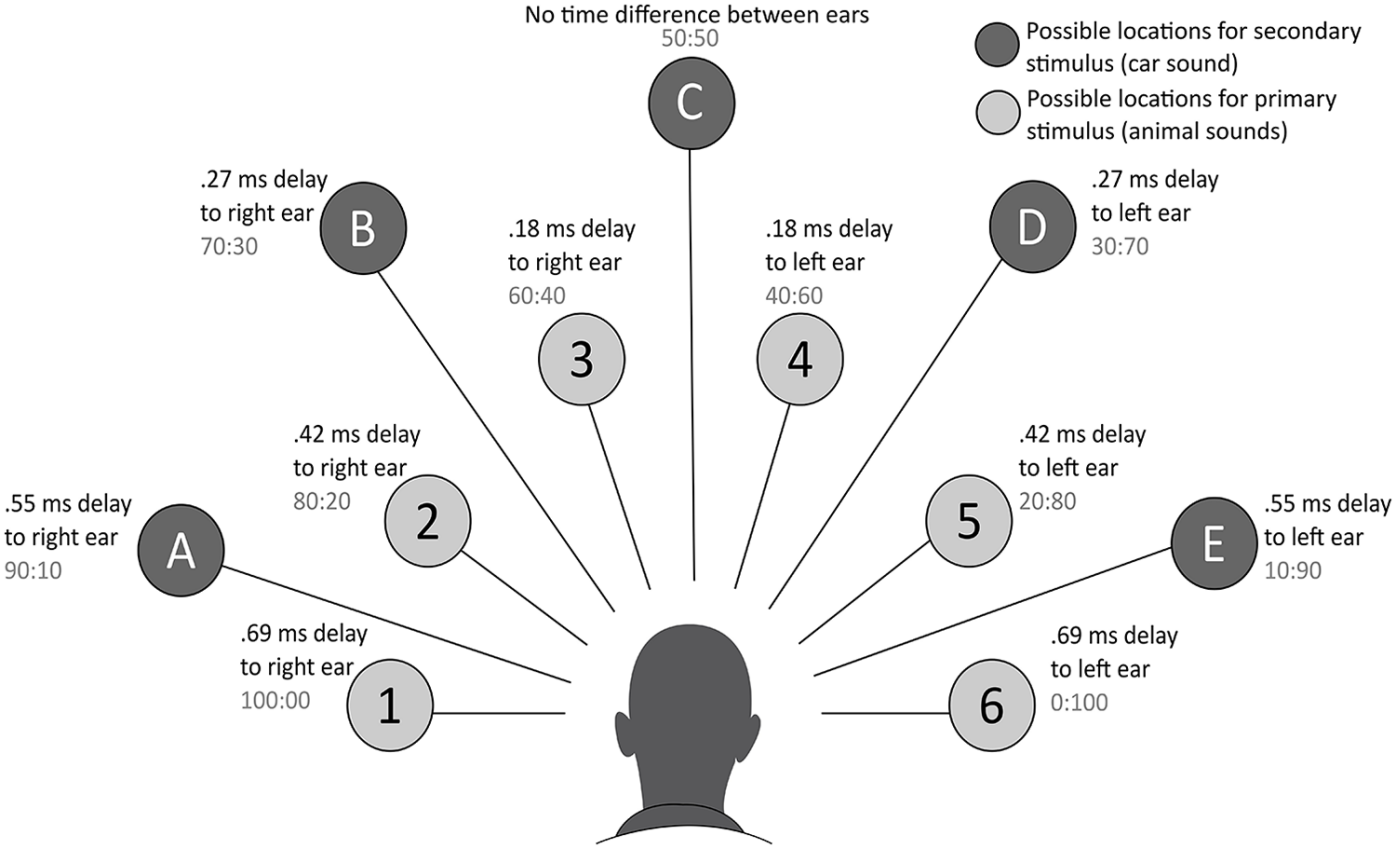
Auditory load task developed by (Fairnie et al., 2016). Possible locations of the animal sounds were placed in the numbered circles 1–6 in the inner ring, whereas circle A–E on the outer ring represents possible locations of the critical stimulus. The sound of the critical stimulus was 9 dB quieter than the sounds of the target stimulus. The milliseconds next to the circles are the interaural time differences, the time the sound needs to travel to the contralateral ear. The ratios (in grey) represent the interaural amplitude difference – the relative amplitude difference between the ears.

The paradigm involved two tasks: a primary auditory search task and a secondary auditory detection task. On the primary task, participants were presented with a number of animal sounds simultaneously, each positioned on an imaginary semi-circle around their head (see Figure 1). On each trial, participants had to indicate which target sound (a dog’s bark or a lion’s roar) was present among other distractor animal sounds (rooster, chicken, crow, cow and a duck) by pressing the corresponding key. The perceptual load was manipulated by changing the number of distractor sounds to create four different load levels: set size 1 where the target was presented exclusively, and set sizes 2, 4 and 6, where in addition to the target, 1, 3 and 5 distractor sounds, respectively, were presented. For the secondary auditory detection task, participants were required to indicate on each trial whether an additional critical stimulus (a car sound) was present or not by pressing the corresponding key. The critical stimulus was presented concurrently with the animal sounds on 50% of the trials, from one of five positions on an imaginary semi-circle around the participant’s head, at a greater eccentricity than the animal. All sounds were presented for a duration of 100 ms and manipulation of interaural differences and amplitude were used to separate the sounds spatially. Participants were given a maximum of 1500 ms to respond but urged to answer as quickly and accurately as possible. This was supported by visual prompts of the dog and lion and a subsequent presentation of the car/no car. It was made explicit to participants that the primary aim was the animal search task, and that this should be prioritised. For full details of audio settings, see Fairnie et al. (2016). Participants started with one practice block in which each set size was presented four times in ascending order. Subsequently participants completed eight blocks of 36 trials (two blocks of each set size). All participants completed the blocks in the same order (set sizes: 2, 4, 1, 6, 6, 1, 4, 2). Participants had the opportunity to take breaks between the blocks. An additional control block was presented at the end of the task and consisted of 64 trials (16 trials per set size) in which participants performed only the secondary task (i.e. had to indicate whether the critical stimulus was present/absent) while ignoring the animal sounds. This was to ensure that the critical stimulus was audible at all levels of load in conditions of full attention, thereby confirming that any failure to detect on the experimental trials was a result of the load manipulation and not due to a general inability to hear the car sound.

Response, accuracy and reaction time (RT) were recorded automatically for each trial. These were used to calculate the following for each participant: primary task accuracy and RT for each level of load, and detection sensitivity on the secondary task for each level of load. The drop in detection sensitivity to the critical stimulus (car sound) from set size 1 to 6 was taken as a measure of perceptual capacity for each person. A large drop in detection sensitivity with increasing in set size indicates that the perceptual load of the central task has taxed the participant’s capacity. Conversely, a smaller drop in detection sensitivity indicates that a participant still has sufficient perceptual capacity available to perform both tasks at the high level of central task load. Therefore, the drop in detection sensitivity on the secondary task allows individual differences in capacity to be established.

### Procedure

Participants completed the SRS-2, the SPQ, the WASI-2, audiometry threshold tasks and the auditory load task in a quiet room with the researcher present. The order of the tasks was counterbalanced and the overall duration of the study was 90 min. At the end of the study, participants were fully debriefed, had the chance to ask questions, and received £10 in cash or as a shopping voucher in return for their participation.

### Data analysis

Scores on the questionnaire measures, and results from the auditory dual-task paradigm, were used to conduct group comparisons between autistic and non-autistic participants. For these analyses, an age- and IQ-matched sub-sample of autistic and non-autistic adults was created (see ‘Results’ section for demographic information). This sub-sample was used to establish whether there were differences in auditory perceptual capacity and sensory sensitivity between the diagnostic groups. Analysis of Variance (ANOVA), *t*-tests or non-parametric equivalents were performed as appropriate. Greenhouse–Geisser corrected values were used where appropriate.

A second set of analyses, using the full sample, were conducted to investigate any association between sensory sensitivity, autistic traits and auditory perceptual capacity – irrespective of diagnostic category. Tests of correlation were performed on SRS-2 scores, SPQ scores and perceptual capacity (as measured by the drop in detection sensitivity from set size 1 to 6).

## Results

### Group comparisons

#### Sample

To ensure appropriate group comparisons could be made, 30 of the non-autistic participants were selected to match the autistic participants on age, gender and IQ (see Table 1). Matching was achieved by removing the younger non-autistic participants and those with the lowest IQ. In addition, nine participants in the non-autistic group showed SRS-2 scores above the clinical threshold of 60 and were therefore excluded from the sample to ensure that participants in the control group were consistent with the non-clinical population (Constantino & Todd, 2003). One autistic participant was removed from the sample, as they did not meet the cut-off for an autism diagnosis on either the SRS or the ADOS.

**Table 1.** Matched sample.

Variable Gender (M: F)	Autistic *n* = 29 *M* (*SD*) Range	Non-autistic *n* = 30 *M* (*SD*) Range	*p-*value
	16:13	16:14	0.89
Age in years	30.03 (8.85) 18–54	27.87 (9.21) 18–53	0.36
FSIQ-2	*118.69* (12) 94–145	114.43 (10.36) 101–142	0.15
SRS	85.93 (23.89)	35.87 (15.67)	<0.001

FSIQ-2: Full-scale IQ-2; SRS: Social Responsiveness Scale, means and standard deviations (*SD*) in parentheses.

#### Primary auditory search task

The accuracy rates and median RTs on the primary task (animal search task) for each group at each level of load are presented in Table 2. RT data were only included for correct trials, and any RT of below 150 ms was considered to be an error. ANOVA revealed a main effect of load on accuracy (*F*(2.53,144.39) = 34.34; *p* < 0.001; $\eta_{p}^{2}$ = 0.38) with increased error rates at higher set sizes. There was no significant main effect of group (*F* < 1) nor interaction between the two variables (*F*(2.53, 144.39) = 1.17, *p* = 0.32, $\eta_{p}^{2}$ = 0.02). There was a significant main effect of load on RT (*F*(2.59,147.75) = 31.05, *p* < 0.001; $\eta_{p}^{2}$ = 0.35). The main effect of group (*F*(1,57) = 1.61, *p* = 0.21, $\eta_{p}^{2}$ = 0.03) and the interaction (*F*(2.59,147.75) = 1.04; *p* = 0.38; $\eta_{p}^{2}$ = 0.02) were not significant. This showed that participants’ accuracy levels decreased and RT increased with higher levels of perceptual load, indicating that the load manipulation was effective. However, overall accuracy levels for the auditory search task were very high, even at set size 6; the accuracy rates were 84%, which suggests that the primary task may not exhausted the perceptual capacity of the participants.

**Table 2.** Mean accuracy and RT on the primary task, and detection sensitivity on the secondary task for each set size by group.

Set size	Accuracy (%)		1. RT (ms)		Detection sensitivity (A)	
	Autistic	Non-autistic	Autistic	Non-autistic	Autistic	Non-autistic
1	94.33 (8.61)	94.96 (5.78)	802.06 (163.98)	773.6 (185.72)	0.90 (0.18)	0.92 (0.07)
2	87.93 (10.06)	84.4 (9.76)	986.01 (212.93)	904.73 (206.91)	0.86 (0.19)	0.88 (0.09)
4	85.37 (13.6)	82.06 (14.39)	970.02 (249.61)	912.03 (238.36)	0.87 (0.19)	0.86 (0.10)
6	84.89 (12.49)	82.43 (12.96)	1015.61 (256.34)	917.80 (258.09)	0.87 (0.11)	0.84 (0.2)

The standard deviations are given in parentheses.

#### Secondary detection task

False alarm and hit rates were used to calculate the detection sensitivity for the critical stimulus. Inspection of the data revealed that false alarms and hits were not normally distributed. Therefore A, the non-parametric equivalent of d’, was used as suggested in Zhang & Mueller, 2005. The detection sensitivity A takes values between 0 and 1, where 1 indicates perfect detection and 0.5 indicates that the signal cannot be distinguished from noise (Stanislaw & Todorov, 1999).

**Figure 2.**
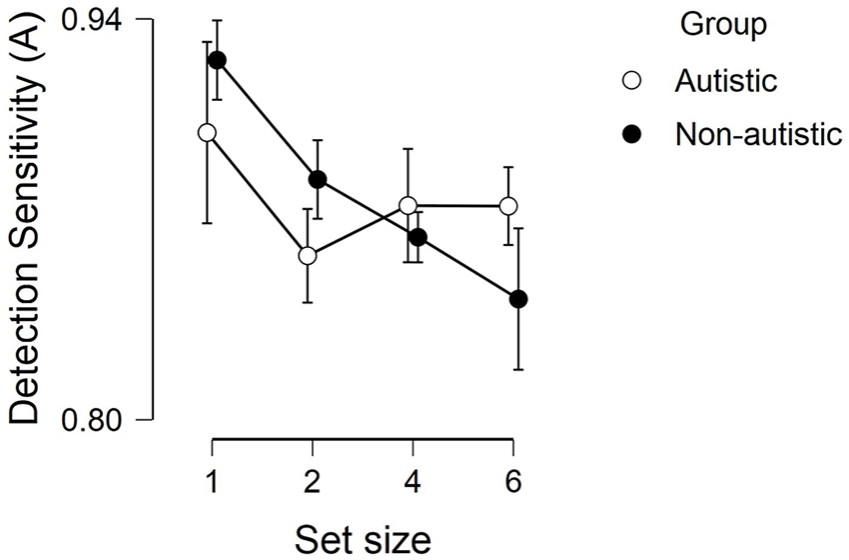
The detection sensitivity (A) for each group at each set size. Note. The figure represents a section of the scale ranging from .75 to 1.0, the error bars represent the standard error of the mean.

The ANOVA indicated a main effect of load on detection sensitivity (*F*(2.19,124.73) = 3.21; *p* = 0.04; $\eta_{p}^{2}$ = 0.05): detection sensitivity for the critical stimulus decreased as the level of load in the primary task increased (see Figure 2). The main effect of group (*F* > 1) and the interaction (*F*(2.19, 124.73) = 1.16, *p* = 0.33, $\eta_{p}^{2}$ = 0.02) were not significant. However, inspection of Figure 2 shows that although the group differences were not significant, there appears to be a steady decline in detection sensitivity for the non-autistic participants, but a plateau for the autistic participants’ detection sensitivity.

#### Control block

On the control block, where participants performed the secondary task without performing the primary task, participants’ ability to detect the critical stimulus was high (>84%) for both groups under the all load conditions.

### Correlation analyses

To test the relationship between sensory sensitivity, perceptual capacity (as measured by drop in detection sensitivity from set size 1 to 6, henceforth A-drop) and autism traits (as measured on the SRS-2), the data were analysed irrespective of diagnostic labels in the full sample of 91 participants (male = 46, female = 45), participants’ age ranged from 18 to 55 (*M* = 27.27 *SD* = 8.6) years and had a mean IQ of 114.7 (*SD* = 12.56, range = 88–145). Participants’ SRS scores ranged from 10 to 130 (*M* = 57.11, *SD* = 29.91). We also entered the *t*-scores for the repetitive and restricted behaviour (RRB) subscale of the SRS-2 into the correlation (*M* = 57.87, *SD* = 14.33, range = 16–90) to establish whether it was specifically the level of non-social autistic traits that would be associated with perceptual capacity. Participants showed a large range of sensory sensitivity scores, ranging from 6 to 90 (where low scores indicate hypersensitivity and high scores indicate hyposensitivity, *M* = 48.09, *SD* = 15.49). As expected, the groups significantly differed on the SPQ score (*t*(58) = 2.25, *p* = 0.03, autism group: *M* = 41.94, *SD* = 17.5; control group: *M* = 50.90, *SD* = 13.65). The mean drop in detection sensitivity (A-drop) from set size 1 to 6 on the auditory task was 0.08 with an *SD* of 0.19 (range −0.75 to 0.91).

**Figure 3.**
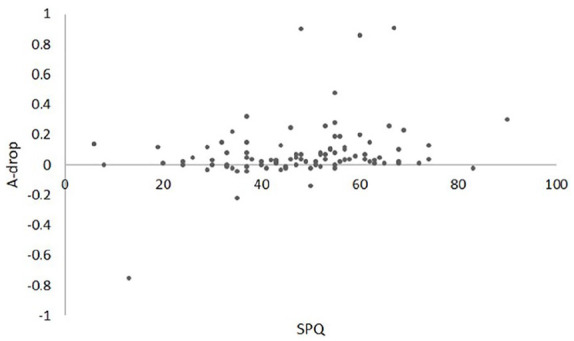
Scatterplot of SPQ score and the descent in detection sensitivity between set size 1 and 6 (A-drop). Note: Negative, constant or small positive values of A-drop from set size 1 to 6 reflect higher perceptual capacity. For the SPQ, the lower scores indicate higher sensory.

The drop in detection sensitivity was not normally distributed; therefore, the non-parametric Spearman’s rho correlations were carried out. Scores on the SPQ were positively correlated with the drop in detection sensitivity (*r*_s_ = 0.25, *p* = 0.018): higher sensory sensitivity was associated with higher levels of perceptual capacity (see Figure 3). There was no significant relationship between perceptual capacity and autistic traits as measured on the SRS-2 (*r*_s_ = −0.13, *p* = 0.22) or between perceptual capacity and the RRB scale (*r*_s_ = −0.103, *p* > 0.05). As expected, SPQ and SRS levels were negatively correlated (*r*_s_ = −0.34, *p* < 0.001), suggesting higher levels of autistic traits were associated with sensory hypersensitivity. Sensory processing was also significantly correlated with RRB (*r*_s_ = −0.35, *p* = 0.001).

### Exploratory cluster analysis

To test the theoretical prediction that sensory hypersensitivity and perceptual capacity would cluster together, an initial hierarchical cluster analysis was carried out. The variables SPQ and A-drop were standardised using z-score transformation to control for unequal scales of the variables. Ward’s (1963) clustering method was employed with the squared Euclidean distance calculation to measure similarity. The analysis revealed two clusters. K-means clustering was carried out with two clusters based on standardised z-scores for the variables. One-way ANOVA of the unstandardised scores confirmed that the groups are significantly different for A-drop (*F*(1,89) = 9.66, *p* = 0.003) and for the SPQ (F(1,89) = 133.7, *p* = 0.001). For mean unstandardised and z-scores of the cluster centres, see Table 3.

**Table 3.** Cluster analysis results: means, standard deviations and cluster centres.

Cluster	A-drop	SPQ	Cluster centres	
			z-score A-drop	z-score SPQ
Cluster 1 (*n* = 47)	0.02 (0.13)	36.23 (10.82)	−0.75	−0.30
Cluster 2 (*n* = 44)	0.14 (0.22)	60.25 (8.81)	0.8	0.32

SPQ: Sensory Perception Quotient.

Cluster 1 included 47 participants with hypersensitity and increased perceptual capacity (a small drop in detection sensitivity). In total, 44 participants in Cluster 2 show levels of hyposensitivity and a larger drop in detection sensitivity. In Tavassoli et al’.s (2014) study, the mean SPQ score for the non-autistic participants was at 43.01 and 38.55 for the autistic participants. Therefore, Cluster 1 with a mean of 36.23 can be considered hypersensitive, and Cluster 2 with a mean of 60.25 can be considered hyposensitive.

## Discussion

In this study, we examined the relationship between a perceptual capacity and sensory sensitivities. We demonstrated that there was an association between higher levels of perceptual capacity (as measured on a computer-based attention task) and self-reported sensory hypersensitivity. A cluster analysis confirmed two patterns: one group of individuals with sensory hypersensitivity and increased perceptual capacity and a second cluster with people demonstrating decreased sensory sensitivity and perceptual processing. With respect to the latter, it seems that having a lower perceptual capacity and corresponding sensory hyposensitivity may more readily render people ‘deaf’ to additional sounds when they are engaged in a task (compared to those with higher capacity and sensory sensitivity). This may become dangerous, for instance, if a driver does not pay to attention to the road because they are distracted by complicated satnav instructions (Raveh & Lavie, 2015). It would be interesting to explore further, whether the individuals in Cluster 2 have experienced any such events.

While the implications of sensory hyposensitivity are important, they appear to have a less severe impact on daily life than sensory hypersensitivity. Indeed, sensory sensitivities can be extremely debilitating for those who experience them. For example, in an online forum, one autistic person remarks that during a sensory overload

. . . [I]can’t think. I can’t find the right word that I know I know. I feel like my mind is in a fog. Things seem spacey, unreal. I feel my head could spin and sometimes I do get dizzy from it. (autismforums.com, 2017)

Although a causal relationship cannot be confirmed from the current study, the association we have identified may begin to highlight the real-life ramifications of increased perceptual capacity and underlying processes that might be involved in sensory hypersensitivity. The over-arousal, which so many autistic people report, may well be a result of taking in more sensory information to fill a higher perceptual capacity.

As well as over-arousal, the higher perceptual capacity also appears to be associated with increased susceptibility to distraction. For instance, having spare capacity can mean noticing a fly in a room while on a conference call or hearing music that is set to the lowest setting while listening to a co-worker, or hearing a dog’s whistle while paying attention to a friend’s stories in the park. Though not as debilitating as over-arousal, being distracted by irrelevant stimuli can undermine our ability to perform many everyday tasks.

Understanding that the negative aspects of sensory processing experienced by many autistic individuals (and indeed some non-autistic individuals) are associated with increased perceptual capacity can facilitate more effective intervention. For example, the way to ameliorate unwanted processing due to higher perceptual capacity appears to be the *addition* of task-relevant information. Forster and Lavie (2007) found that for those more susceptible to distraction (as measured by high scores on the Cognitive Failures Task (CFT, Broadbent et al., 1982), increasing the perceptual load of the task they were trying to perform was useful in maintaining focus. On a low load task, they found that participants with high CFT scores showed more distractor interference than those with low CFT scores. Raising the perceptual load of the task, however, eliminated the impact of distractors for both groups of participants.

This work, together with the results of this study, suggests that while care must be taken to avoid over-arousal, presenting task-relevant or non-competing information may help fill spare capacity in such a way that the additional processing does not lead to sensory distress or distract someone from the task at hand. Preliminary evidence of the value of such an approach in the classroom can be seen in our prior research with autistic and non-autistic young people, for whom the addition of task-relevant information allowed additional perceptual capacity to be harnessed (Remington et al., 2019).

Despite the autistic participants reporting significantly more sensory sensitivities than their non-autistic peers, in this study, we did not find an association between autistic traits (as measured by SRS scores) and perceptual capacity. This is interesting, given the previous research on increased perceptual capacity in autism, and suggests that individual differences in capacity may be related to sensory processing irrespective of diagnostic label. This also raises the question of whether increased perceptual capacity and sensory hypersensitivities might be found in other subgroups of the population. For example, altered attention has been found in those with anxiety disorders. Berggren and colleagues (2015) showed that participants with high trait anxiety also showed higher levels of perceptual capacity. This is in keeping with observations that anxiety is linked to hypervigilance of one’s surrounding (Berggren & Derakshan, 2013; Eysenck et al., 2007) and is especially interesting, as studies have also found a direct association between sensory processing atypicalities and anxiety in autistic and non-autistic adults (Horder et al., 2014). In addition, future research should also explore developmental conditions, other than autism, which are associated with sensory processing atypicalities, such as attention-deficit hyperactivity disorder (ADHD; Ghanizadeh, 2011) and Williams Syndrome (Engel-Yeger et al., 2011). Understanding the role of increased capacity in these conditions may, as with autism, inform interventions for those struggling with sensory sensitivities.

The current results raise the interesting question of whether increased perceptual capacity and sensory hypersensitivities extend beyond those on the autistic spectrum. It is important to note, however, that the lack of significant correlation between SRS scores and perceptual capacity in the current study may also be an artefact of our participant sample. In this study, we observed higher perceptual capacity in the non-autistic participants compared to previous studies (Fairnie et al., 2016; Remington & Fairnie, 2017). Given that the performance of the autistic participants was in line with the previous studies, this resulted in equivalent performance on the auditory dual-task paradigm with no significant differences in perceptual capacity when group comparisons were conducted. As all participants’ performance was extremely high across the various set sizes, capacity may not have been exhausted for either groups – even under the highest level of perceptual load. To fully expose any group differences, it is necessary to raise the level of central task load until it fills the perceptual capacity (as evident by an ability to successfully perform the secondary task) of at least one group of participants (or establish that the same level of load fills the capacity of both). It may be necessary, therefore, to extend the difficulty of the current task (by adding additional set sizes) to more conclusively determine whether there is an association between perceptual capacity and autistic symptomatology. Indeed, even though the group differences were not significant, inspection of the graph in Figure 2 shows a steady decline in detection sensitivity for the non-autistic participants, but a plateau beyond set size 4 for the autistic participants.

The SPQ used in this study investigates sensory discrimination abilities. While behavioural responses related to sensory experiences are presently not assessed in the SPQ, further research might investigate sensory reactivity in relationship to perceptual capacity.

In sum, the findings of the current study have demonstrated the link between increased auditory perceptual capacity and sensory sensitivities experienced by individuals on a daily basis. This highlights not only the relevance of increased perceptual capacity to the lived experiences of autistic and non-autistic individuals but also offers a targeted approach to intervention to support those who experience the negative impact of these traits. Indeed, sensory symptoms associated with autism have been shown to negatively impact on many areas of life – often more so than social communication challenges. For example, Ashburner et al. (2008) found that estimated intelligence does not predict academic performance, but sensory seeking behaviours, hyposensitivity and auditory filtering were negatively associated with academic performance for autistic children, thereby highlighting the important role of sensory experience. Other settings, such as the physical environment in the workplace, can also be challenging. Lighting, temperature, sounds and smells have been flagged up as potential sources of sensory overload and distraction that negatively impact on employment outcomes (Robertson, 2012). As such, our study has important practical implications for aspects of daily life, and we hope the findings can be used to inform approaches to effectively manage individual differences in attention and perception within therapy, education and employment.
